# Mental Flexibility Influences the Association Between Poor Balance and Falls in Older People – A Secondary Analysis

**DOI:** 10.3389/fnagi.2019.00133

**Published:** 2019-06-12

**Authors:** Frederico Pieruccini-Faria, Stephen R. Lord, Barbara Toson, Wolfgang Kemmler, Daniel Schoene

**Affiliations:** ^1^Geriatric Division, Department of Medicine, University of Western Ontario, London, ON, Canada; ^2^Falls, Balance and Injury Research Centre, Neuroscience Research Australia, University of New South Wales, Sydney, NSW, Australia; ^3^Institute of Medical Physics, Friedrich–Alexander University Erlangen–Nürnberg, Erlangen, Germany

**Keywords:** accidental falls, aged, balance, cognition, executive functions, mental flexibility

## Abstract

Impairments of balance predispose older people to falls. Some cognitive functions, especially executive functioning have been shown to affect balance and discriminate fallers from non-fallers. Mental flexibility is a component of the executive function and comprises multiple cognitive processes that work together to adjust the course of thoughts or actions according to the changing demands of a situation without the use of explicit instructions. However, the role of mental flexibility in balance in older people remains unclear. The study aim was to examine the relationship between mental flexibility and falls in a cohort of 212 older people (80.6 ± 4.9 years; 62% female). We hypothesized that: (i) participants with impaired balance would have worse mental flexibility compared to those with good balance; and (ii) poor mental flexibility would predict falls in the sub-group with impaired balance. Balance performance was assessed by measuring postural sway while standing on a medium density foam mat with eyes open for 30 s. Mental flexibility was assessed using a computerized short-form of the Wisconsin Card Sorting Test (WCST; 64 cards) with its sub-components comprising general performance, perseveration, failure-to-maintain set and conceptual ability. Falls were measured prospectively for 12-months using monthly calendars. MANCOVA revealed that WCST performance was associated with balance [Wilks’ Lambda = 0.883, *F* = 2.168; *p* = 0.013, partial eta squared ($\eta_{p}^{2}$) = 0.061] due primarily to reduced concept formation ability [*F*_(2,207)_ = 5.787, *p* = 0.004, $\eta_{p}^{2}$ = 0.053]. Negative binomial regression analysis adjusting for age, education, contrast sensitivity, proprioception, inhibition, and inhibitory choice stepping reaction time (iCSRT) revealed that lower concept formation ability was predictive for falls [Incidence Rate Ratio 1.048 (95% confidence interval 1.016–1.081)]. Further, lower concept formation ability partly explained the association between balance and falls: i.e., fallers in the upper balance tertile had reduced concept formation performance whereas non-fallers had similar concept formation performance across the three balance tertiles. These findings suggest that poor mental flexibility affects the ability to maintain steady balance contributing to increased risk of falls in older people.

## Introduction

There is growing evidence for interrelationships between balance and cognition in older people and their associations with falls (Schäfer et al., 2006; Yogev-Seligmann et al., 2008). Impaired balance has been consistently identified as a risk factor for falls in older people (Lord et al., 2007), with objective balance measures being good markers of fall risk that can be easily administered in clinical practice (Horak et al., 1989; Piirtola and Era, 2006; Sturnieks et al., 2011). It has been shown that standing balance requires cognitive resources (Teasdale and Simoneau, 2001; Horak, 2006), and older people use cognitive control to regulate sensorimotor processing during balance tasks (Redfern et al., 2001, 2004). Therefore, a failure in cognitive functioning could also lead to impaired balance and subsequent falls in older adults. Especially executive functions have been associated with increased fall risk in older people (Anstey et al., 2009; Hsu et al., 2012; Mirelman et al., 2012).

Executive functions comprise higher-order cognitive processes responsible for planning, controlling and coordinating complex cognitive tasks (Miyake et al., 2000), although no consensus definition has been agreed upon. Impaired executive functions, especially inhibition, have been identified as risk factors for falls in several studies. Mirelman et al. (2012) demonstrated that poor executive function, especially inadequate response inhibition, predicts falls in well-functioning older people, and Anstey et al. (2009) found subtle differences in inhibition are an early marker of increased fall risk, while multiple cognitive domains are impaired in recurrent fallers. We have also shown that slow inhibitory choice stepping reaction time (iCSRT), a test of combined inhibition and rapid step initiation, was predictive of falls in older people and that this association was not mediated by body sway, processing speed or attention (Schoene et al., 2017).

Mental flexibility is a component of executive functioning, but without an agreed definition as well (Ionescu, 2012). Here, it is defined as a construct composed of multiple cognitive processes that work together to adjust the course of thoughts or actions according to the changing demands of a situation without the use of explicit instructions (Berg, 1948). However, depending on definitions, cognitive processes including abstract reasoning, concept formation (understanding implicit task rules), inhibition of irrelevant information, task switching, set shifting and working memory (information updating) comprise the mental flexibility construct (Ionescu, 2012), functions that partially overlap. Mental flexibility is important to shift mental sets to adjust behaviors quickly, e.g., when (unpredictable) environmental demands require proactive and/or reactive responses to avoid a loss of balance. However, to our knowledge no study to date has investigated the effect of mental flexibility on balance and fall risk in older people.

In the above studies on executive functions and falls, relatively easy reaction time tasks for executive functions have been administered for which the task rules were clear to participants. For elucidating the importance of mental flexibility, i.e., abstract reasoning and concept formation for fall risk in older adults, more complex tasks are required for which participants have to implicitly learn the task rules. Previous studies have also not investigated the role of mental flexibility as a confounder or potential effect modifier in the association between balance control and falls in older people. Obtaining insights into these processes would increase our understanding of the complex inter-relationships between cognitive and motor functions and their impact on falls.

The objective of the current study, therefore, was to investigate the importance of more complex mental flexibility components to balance control and falls in older people and their role as potential confounders and moderators in the association between poor balance and falls. We hypothesized that: (i) participants with impaired balance would have worse mental flexibility compared to those with good balance; (ii) poor mental flexibility would predict falls in the sub-group with impaired balance.

## Materials and Methods

This is a planned secondary analysis of a cohort study conducted between July and December 2012 on stepping, cognition and falls in 212 independently living older adults in Sydney, Australia (Schoene et al., 2017). The study was conducted according to the Declaration of Helsinki and was approved by the University of New South Wales Human Research Ethics Committee (HREC 11159). All participants gave written informed consent prior to data collection.

### Participants

Participants were recruited via a research institute’s volunteer database and from residents of a retirement village in Sydney, Australia. The following inclusion criteria were applied: (1) aged 70 years and older; (2) ambulant with or without walking aids; and (3) able to step without assistance (step size 25–30 cm). People were excluded if they were cognitively impaired [diagnosis of neurodegenerative disease, Rapid Dementia Screening Test ≤4 (Kalbe et al., 2003)], had movement disorders (self-reported) or limiting lower limb pain and visual impairments that could not be corrected [>6/16 on a LogMAR visual acuity chart, color-blind]. Experienced research staff performed the screening, assigned participants to the study, conducted the assessments and informed participants about the follow-up procedures.

### Balance Assessment

Sway path in millimeters was measured with a sway-meter that measured displacements of the body at the level of the waist. Testing was performed with participants standing on a medium density foam rubber mat (65 × 65 × 15 cm thick) with eyes open for 30 s (Lord et al., 2003). This device has demonstrated reliability and validity for measuring sway under multiple sensory conditions (Sturnieks et al., 2011). The sample was split into tertiles: good balance (62 to 146 mm, *N* = 71), fair balance (147 to 209 mm, *N* = 72), and poor balance (>210 mm, *N* = 69).

### Wisconsin Card Sorting Test Short-Form (WCST)

Mental flexibility was assessed using the Wisconsin Card Sorting Test (WCST). This test measures implicit rule learning, abstract reasoning and set-shifting. In this test, participants matched stimulus cards according to a rule (color, shape, or number) without being informed of the rule. After successful understanding of the rule (10 correct matches), the rule is changed to enforce a shift in cognitive sets. After each trial, visual feedback was displayed for 500 ms. Four general executive processes are measured with the WCST: (1) *General Performance*: total correct responses; (2) *Perseveration*: Perseverative responses or perseverative errors (inability to use feedback from previous errors to produce correct behaviors – only perseverative responses that are also errors); (3) *Failure to maintain set*: the number of times the participant fails to make between five to nine correct responses in a row; (4) *Conceptual Ability*: number of trials needed to complete the first category (concept formation) and proportion (in %) of consecutive correct responses occurring in runs of three or more (conceptual level responses) – this ability measures how many trials a participant takes to understand the implicit task rule to switch to a new mental set (i.e., new rule) (Haaland et al., 1987). A WCST short form (64 trials) was computer-administered using a freely available and validated software tool [The Psychology Experiment Building Language (PEBL), version 0.14^1^] (Piper et al., 2012; Fox et al., 2013).

### Attentional Network Test (ANT)

The ANT combines cued reaction time and flanker tasks and requires participants to signal by button press whether a central arrow points left or right (Fan et al., 2002). The arrow appears above or below fixation and may or may not be accompanied by flankers which can emerge in congruent or incongruent form. Scores for three attentional networks are generated by measuring how response times are influenced by alerting cues, spatial cues, and flankers. The latter is used to assess the efficiency of the executive network (inhibitory control) and requires individuals to quickly suppress their prepotent response in order to resolve conflicts correctly by indicating the direction of congruent and incongruent flankers. It is calculated by subtracting the mean RT of all congruent flanking conditions, summed across cue types, from the mean RT of incongruent flanking conditions (Fan et al., 2002). We used this score as a measure of inhibition and to determine if measures of WCST measure a different component of executive functioning.

### Inhibitory Choice Stepping Reaction Time (iCSRT)

The iCSRT is a development of the CSRT (Lord and Fitzpatrick, 2001; Schoene et al., 2011, 2017). Participants were asked to stand on the two stance panels. Stance and target panels were displayed on the screen. For “go” trials, participants were instructed to step onto a panel as quickly as possible when the corresponding arrow on the monitor changed color from white to green. For no-go trials (33% of trials), signaled by an arrow on the screen changing from white to purple, participants had to suppress the prepotent stepping response. Stimuli were presented for 100 ms and time between trials was randomized, occurring 0.5 to 1 s after returning both feet to stance panels. Immediate visual feedback of step completion was provided. After six practice trials (one stimulus for each target panel with two stimuli to be withheld), a random sequence of 36 trials was presented. For this secondary analysis we used the time in milliseconds for step RT (iCSRT-RT) as fall-related measure of executive functioning.

### Additional Measures

Socio-demographic and medical information was collected by self-report questionnaires. The 12-item World Health Organization Disability Assessment Schedule (WHODAS) 2.0 was used as a generic assessment of health and disability; participants reported their level of impairment for several instrumental activities of daily living on a five-point Likert scale ^2^. To record the number of comorbidities we used the Functional Comorbidity Index for which participants are required to report whether they had one or more of 18 chronic conditions and from which a sum score was calculated (Groll et al., 2005). Depressive symptoms were measured using the nine-item Patient Health Questionnaire (PHQ-9) (Kroenke et al., 2001). Concern about falling was measured by the iconographical falls-efficacy scale (Icon-FES), a questionnaire which depicts line drawings of a person undertaking a range of simple through to more demanding activities of daily living (Delbaere et al., 2011). Sum scores for the PHQ-9 and Icon-FES were calculated with high scores indicating increased depressive symptoms and concern about falling, respectively.

Sensorimotor performance was assessed as part of the Physiological Profile Assessment, comprising four tests in addition to postural sway (Lord et al., 2003):

Contrast sensitivity was assessed with the Melbourne Edge Test (Verbaken and Johnston, 1986), using a chart comprising of 20 circular test patches (25 mm diameter), with a series of edges of reducing contrast and of variable orientation;
Proprioception was measured with a lower limb matching task. Errors in degrees were recorded on a protractor inscribed on a vertical clear acrylic sheet (60 × 60 × 1 cm) placed between the legs;
Maximum isometric knee extension strength was measured in the dominant leg while seated with a knee angle of 90 degrees;
Simple reaction time was recorded using a randomly presented light stimulus and a finger-press as the response.

### Falls

Falls were monitored prospectively over 12 months with daily falls calendars that were sent back monthly using pre-paid envelopes. If calendars were not returned, follow-up phone calls were made within 14 days of end of each month to remind participants to send back their calendars and to obtain additional information on falls. A fall was defined as “*unintentionally coming to the ground, floor or lower level*” (Lamb et al., 2005). People that experienced at least one fall in the follow-up period were classified as fallers.

### Statistical Analysis

Missing data (less than 2%) were imputed using the Expectation Maximization method (Little’s missing completely at random test *p* > 0.05). Sample characteristics were compared using Analyses of Variance (ANOVAs) and chi squared tests. Based on previous study findings, showing that contrast sensitivity, proprioception, hand reaction time, and quadriceps strength were associated with balance control of older people while standing on a medium-density foam mat (Lord and Ward, 1994), balance performance was compared between groups applying Analysis of Covariance (ANCOVA) adjusting for these variables and other covariates we considered necessary to control for following comparison of demographic and sensorimotor data between the groups (Table 1). As sub-scores of the WCST are highly correlated (also observed in the current study – Supplementary Table S1), and are associated with both age and education level (Haaland et al., 1987; Barkley, 1997), multivariate analysis of covariance procedures (MANCOVAS) were performed to compare groups, with significant differences in Wilk’s Lambda used to determine whether mental flexibility was different between groups. Univariate ANOVAs were then applied to determine which variable of the WCST differed between groups using Bonferroni adjustments. (Partial) Eta squared (η^2^/$\eta_{p}^{2}$) was calculated as effect size. Univariate and multiple negative binomial regression analyses (with individual follow-up time in months used as offset variable) were applied to determine the associations between balance as categorical variable (tertiles; good balance as reference category) and WCST and fall rate (number of falls; capped at 10 falls). Incidence rate ratios (IRR) with 95% confidence intervals were calculated. WCST performance was also investigated as a potential confounder in the association of balance and fall risk using a 10% change-in-estimate criterion as an indicator of a confounding effect (Budtz-Jorgensen et al., 2007). Potential effect modification by WCST performance was determined using interaction terms. The level of significance was set to 5%. Analyses were performed with SPSS for Windows (version 25, IBM).

**Table 1:** Demographics, health, psychological, sensory, and fall risk profile of participants stratified by balance performance.

Mean (SD)	Good balance (*N* = 71)	Fair balance (*N* = 72)	Poor balance (*N* = 69)	*P*-value	Eta squared
Age (years)	80.0 (4.8)	79.9 (4.9)	81.9 (4.9)	**0.023**	0.035
Sex (number, % female)	46 (65)	46 (64)	39 (57)	0.544	
Years of education	12.1 (3.6)	12.4 (3.3)	12.6 (3.8)	0.725	0.003
Height (cm)	163.3 (8.1)	162.6 (9.0)	162.0 (8.5)	0.650	
Weight (Kg)	73.4 (16.1)	71.4 (12.2)	71.5 (13.6)	0.622	
Contrast sensitivity (dB)	21.0 (1.4)	21.5 (1.5)	20.8 (1.7)	**0.022**	0.036
Knee extension strength (kg)	28.9 (11.5)	25.8 (8.7)	26.8 (8.7)	0.152	0.018
Proprioception (°)	1.9 (1.4)	1.9 (1.2)	2.6 (1.7)	**0.006**	0.049
Hand reaction time (ms)	230 (35)	247 (47)	245 (50)	**0.044**	0.030
Falls past year (% yes)	35	40	57	**0.030**	
Concern about falling (10–40)^∗^	16.7 (4.5)	17.9 (4.9)	18.1 (4.8)	0.169	
Depression (0–27)^∗^	2.1 (2.4)	2.3 (2.8)	2.6 (3.0)	0.927	
Comorbidity Index^∗^	3.3 (2.3)	3.5 (2.2)	3.7 (2.3)	0.590	
ANT executive network (ms)	110 (55)	97 (46)	122 (87)	*0.080*	0.024
Inhibitory choice stepping reaction time (ms)	866 (96)	864 (105)	886 (145)	0.475	0.007

*Variables are presented in mean (SD) unless otherwise stated. Significant results from univariate tests are shown in bold; trend for significance in italic. ^∗^higher values indicate larger levels of the construct; Fear of falling was assessed using the Icon-FES; Depression was assessed using the PHQ-9; Comorbidity Index was measured using the Functional Comorbidity Index providing a sum score of 18 chronic conditions.*

## Results

Participant characteristics are displayed in Table 1. Consistent with previous findings, those with poor balance were older and performed worse in tests of contrast sensitivity, proprioception and hand reaction time than those with good balance (Lord and Ward, 1994). In contrast, knee extension strength did not differ among the balance groups in this study. A trend for significance was observed for the ability to resolve conflict (inhibition-ANT executive network), with individuals in the fair balance group being better than those in the poor balance group (*p* = 0.025).

### Balance

As expected, sway paths differed among the three balance groups in univariate analysis [*F*_(2,209)_ = 100.695, *p* < 0.001, η^2^ = 0.491]. After controlling for age, contrast sensitivity, proprioception, knee extension strength, simple reaction time and ANT performance, significant differences remained [*F*_(2,203)_ = 80.861, *p* < 0.001, $\eta_{p}^{2}$ = 0.446] (Table 2), with Bonferroni *post hoc* tests showing linear differences between each tertile.

**Table 2:** Unadjusted (ANOVA) and adjusted (ANCOVA) analyses for the association between postural sway and balance tertiles.

Models	Comparisons	*F*	*p*-value
**Base model**	Balance tertiles	100.695	**<0.001**
**Model 1**	Balance tertiles	80.861	**<0.001**
	Age	4.556	**0.034**
	Contrast sensitivity	4.665	**0.032**
	Knee extension strength	1.331	0.250
	Proprioception	4.917	**0.028**
	Reaction time	0.046	0.830
	ANT executive network	0.159	0.691

*The ANCOVA model included current and previously reported factors associated with postural sway in older adults. Significant results from univariate tests are shown in bold.*

### Mental Flexibility

The MANCOVA, controlling for age and education, revealed the balance groups had different cognitive flexibility profiles [Wilks’ Lambda = 0.883, *F* = 2.168; *p* = 0.013, $\eta_{p}^{2}$ = 0.061]. *Post hoc* univariate ANOVAs revealed the difference lied in the number of trials required to complete the first category [*concept formation*, *F*_(2,207)_ = 5.787, *p* = 0.004, $\eta_{p}^{2}$ = 0.053]. Bonferroni-adjusted pairwise comparisons showed the poor balance group required more trials than the good balance group (*p* = 0.003) (Table 3). Comparing upper and lower balance group tertiles, there was also a trend indicating the poor balance group gave fewer correct conceptual responses compared to the good balance group [44.7 vs. 47.3%, respectively, *F*_(1,136)_ = 3.581, *p* = 0.05].

**Table 3:** Mean and standard deviations of Wisconsin Card Sorting Test (WCST) sub-components.

WCST scores, mean (SD)	Good balance (*N* = 71)	Fair balance (*N* = 72)	Poor balance (*N* = 69)	*F*	df	*P*-value	Partial Eta squared
Total errors	12.5 (3.6)	12.7 (4.5)	14.0 (4.9)	1.633	2, 207	0.379	0.009
Perseverative responses (%)	20.0 (4.0)	19.7 (3.8)	20.3 (4.4)	0.201	2, 207	0.650	0.004
Perseverative errors	8.1 (2.7)	7.8 (2.5)	8.8 (3.2)	0.931	2, 207	0.188	0.016
Trials to complete first category	12.2 (3.3)	13.5 (4.6)	15.7 (9.0)	9.157	2, 207	**0.004**	0.053
Conceptual level responses (%)	47.3 (5.3)	47.2 (6.5)	44.7 (7.4)	3.581	2, 207	0.136	0.019
Failure to maintain set	0.63 (0.9)	0.72 (0.9)	0.93 (1.0)	1.426	2, 207	0.212	0.015

*(1) MANCOVAS were used to compare mental flexibility performance of groups controlling for age and education and Bonferroni p-value adjustments were applied. (2) Significant results from univariate tests are shown in bold.*

### Falls

Two-hundred-seven participants (97.6%) completed the 12-month follow-up for falls. During the 2507 person-months of follow-up (mean 11.8 months), 93 participants (44%) experienced at least one fall. The proportion of fallers differed between groups, with 35% of the good balance group, 40% of the fair balance group, and 57% of the poor balance group reporting at least one fall. The mean number of falls for the good balance group was 0.65 (*SD* 1.17), for the fair balance group 1.01 (1.63) and for the poor balance group 1.74 (*SD* 3.92). Negative binomial regression analysis revealed that balance status (sway tertiles) was associated with an increased rate of falls. Using the good balance group as the reference, the fall rate for both, the fair and poor balance groups increased [IRR 1.91 (1.14–3.22), *p* = 0.015 and IRR 2.80 (1.68–4.65), *p* < 0.001, respectively].

**FIGURE 1:**
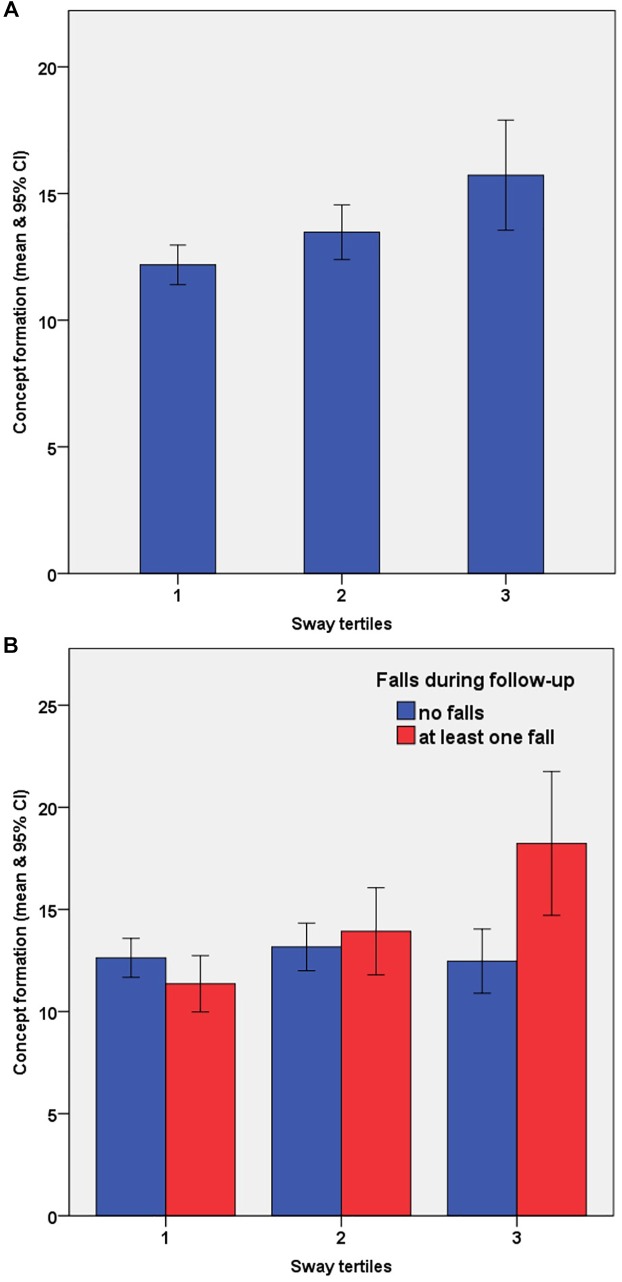
Concept formation ability as measured by the number of trials needed to shift first set during the short version of the Wisconsin Card Sorting Test (WCST) according to balance (sway tertiles) and faller status (one or more falls). Panel **(A)** shows a decline in concept formation performance across sway tertiles; Panel **(B)** shows the additional impact of faller status. Across sway tertiles the concept formation ability did not differ in non-fallers (blue). However, with decreasing balance status, fallers had greater difficulties to implicitly understand the first rule (red).

Concept formation was also associated with an increased fall rate after adjusting for age and education [IRR 1.06 (1.03–1.09), *p* < 0.001]. With respect to the balance tertiles, concept formation predicted falls in the poor balance but not in the fair or good balance groups with the incidence rate in the opposite direction for the good balance group [poor balance: IRR 1.06 (1.02–1.09), *p* = 0.002; fair balance: IRR 1.06 (0.98–1.14), *p* = 0.170; good balance: IRR 0.89 (0.75–1.05), *p* = 0.172]. When entering both variables along with age, education in a multiple negative binomial regression model (enter method), balance status remained significant but was reduced by 13% for the fair balance group and by 24% for the poor balance group (Table 4). Adding contrast sensitivity and proprioception further reduced the effect of balance status with no change in the predictive value of concept formation (model 3, Table 4). This confounding was driven by notably poorer concept formation performance in the fallers and no corresponding reduction in the non-fallers within the poor balance group (Figure 1). Finally, when ANT executive network performance and iCSRT_RT were entered into the model, balance status (tertile 3), concept formation, contrast sensitivity and iCSRT_RT remained significant predictors of falls status (model 4, Table 4), with no evidence of an effect modification by balance status × concept formation (*p* = 0.250). ANT executive network performance was not associated with falls in this sample.

**Table 4:** Unadjusted and adjusted models for the association between balance status and fall rates with 95% confidence intervals.

	IRR model 1	IRR model 2	IRR model 3	IRR model 4
**Balance status**				
Poor balance	2.797 (1.684–4.645)^∗∗∗^	2.124 (1.226–3.680)^∗∗^	1.983 (1.134–3.466)^∗^	2.076 (1.168–3.690)^∗^
Fair balance	1.913 (1.136–3.221)^∗^	1.658 (0.974–2.823)^∧^	1.519 (0.884–2.610)	1.360 (0.772–2.396)
Good balance	1	1	1	1
Conceptual ability		1.051 (1.020–1.082)^∗∗^	1.053 (1.022–1.084)^∗∗^	1.048 (1.016–1.081)^∗∗^
Education		1.075 (1.015–1.138)^∗^	1.079 (1.018–1.143)^∗^	1.105 (1.040–1.174)^∗∗^
Age		0.980 (0.937–1.025)	0.995 (0.949–1.043)	0.976 (0.929–1.026)
Contrast sensitivity			1.193 (1.035–1.375)^∗^	1.226 (1.055–1.426)^∗∗^
Proprioception			1.051 (0.913–1.211)	1.059 (0.917–1.224)
ANT executive network				1.000 (0.997–1.004)
iCSRT_RT				1.003 (1.001–1.005)^∗∗^

*IRR, incidence rate ratios; ^∗^p < 0.05, ^∗∗^p < 0.01, ^∗∗∗^p < 0.001, ^∧^p < 0.10. Balance status, groups categorized by sway tertiles; conceptual ability, number of trials required to shift first set in WCST; contrast sensitivity, Melbourne Edge Test; Proprioception, Knee joint position sense; iCSRT_RT, reaction time component of inhibitory choice stepping reaction time task, a test of combined inhibitory control and rapid step initiation.*

## Discussion

This study found significant associations between cognitive functioning, balance and falls in older people without major cognitive impairments. Consistent with previous findings, poor balance was associated with an increased risk of falling (Piirtola and Era, 2006; Deandrea et al., 2010). Individuals with reduced balance control also performed worse in the computerized short version of the WCST, a test of mental flexibility, mainly due to poor concept formation. Specifically, the poor balance group required more than three additional trials to shift the first mental set demonstrating an impaired ability to implicitly learn a rule. Further analyses demonstrated that this conceptual ability impairment statistically confounded the association between balance status and falls. Compared to people with good balance, those with impaired balance were at increased risk of falling when their concept formation was also poor.

Learning rules of abstract concepts is not obviously related to causes of falling in everyday life. Confirming our second hypothesis, we found older people with impaired balance and poor mental flexibility were more likely to fall. Our findings may indicate fallers have poorer executive function in general, rather than specific mental flexibility deficits confirming results from cognitive and brain-imaging studies that demonstrate executive functions, including mental flexibility reflect some common, and distinct components (Miyake et al., 2000; Wager et al., 2005). It has been previously reported that older people are susceptible to falls if they perform poorly in inhibition tasks (Anstey et al., 2009; Mirelman et al., 2012; Schoene et al., 2017). It has also been demonstrated that poor concept formation is due to lack of inhibition, rather than reduced ability for abstraction (Hartman and Stratton-Salib, 2007). In the current study inhibition was assessed with the executive network of the Attentional Network Test, which requires participants to suppress their prepotent response in order to correctly indicate the direction of congruent in incongruent (conflict resolution) Flanker tasks (Fan et al., 2002). We found a trend that the performance in this task was associated with balance, indicating the relevance of inhibitory processes on balance control. This is in accordance to recent findings that demonstrate the importance of perceptual inhibition for sensory integration during standing balance tasks in older people (Redfern et al., 2018). However, we did not identify a meaningful contribution of this task to falls in older people. This may indicate compensatory processes in our relatively healthy sample without major cognitive impairments and sufficient physical function to do the tests involved in this study. Similarly, we have previously found that a stepping conflict resolution task could not discriminate faller groups whereas the iCSRT (response selection) could (Schoene et al., 2017). The rate of commission errors during the iCSRT task would have been a more genuine reflection of inhibition. However, response selection and response inhibition should not be separated as they are linked with each other in that first the automatic response is inhibited to enable a switch to the controlled response selection (Mostofsky and Simmonds, 2008). The WCST is a demonstrated test of executive function (Greve et al., 2005) and provides distinct information in neuropsychological assessments (Greve et al., 1998). Our finding that concept formation and iCSRT are significant and independent risk factors for falls, implies that more than one executive network influences fall risk. Importantly, this may indicate that both speed dependent (based on reaction time tasks) and non-speed dependent (based on implicit rule learning, without a reaction time component) executive functions are important in this regard.

The interrelationships between sensory, motor, and cognitive functions become stronger with increasing age (Schäfer et al., 2006), and balance and executive functions are inter-connected during standing tasks (Redfern et al., 2009; Taylor et al., 2017). Confirming our first hypothesis, we found that people in different balance tertiles also differed in their concept formation ability. This suggests that understanding an implicit rule may be a sensitive marker of impaired body sway. It is possible that individuals with better concept formation are more efficient in gathering sensory information that signal when corrections in the center of mass displacement are necessary. However, according to the benchmark values reported by Cohen (1988) this can be considered a small effect. This is not surprising as balance depends on multiple sensory, motor, and cognitive functions. Particularly, in individuals with poor balance, worse mental flexibility was associated with more falls, further confirming our second hypothesis. Relative to the good balance reference group, the confounding effect was larger in the poor balance compared to the fair balance group, demonstrating increased cognitive-motor interrelationships in older people with reduced levels of functioning.

In related research conducted with nursing home residents, more than 50% of falls were due to incorrect weight shifting and poor obstacle negotiation (Robinovitch et al., 2013). These postural challenges require the integration of feedback for correct modulation of motor responses and it has been reported age-related declines in WCST are associated with inefficient feedback utilization (Fristoe et al., 1997; Ashendorf and McCaffrey, 2008). This in turn, is mostly due to poor working memory and slow processing speed (Fristoe et al., 1997), which are cognitive domains associated with falls in older people (Anstey et al., 2009). Moreover, anticipation, sensory perception, and cognitive processing of potential balance threats require cognitive resources and this is particularly the case for older adults (Rankin et al., 2000; Bernard-Demanze et al., 2009). Situational awareness, the understanding of factors that contribute to optimal task performance in expected and unexpected conditions, also affects these factors (Caserta and Abrams, 2007), i.e., a quick appraisal of the individual-environment interaction facilitates protective postural responses (Melzer et al., 2001). Indeed, findings from the current study demonstrate that non-fallers react faster in a complex stepping task (Schoene et al., 2017). Implicit rule learning may be important for navigating through complex environments and older fallers may be too slow and/or too inaccurate to process the required information. Reduced mental flexibility may compromise the ability to use feedback from the body and environment to modulate center of mass displacement required to respond appropriately to internal or external changes.

Our findings may in part explain the poorer performance of balance-impaired people in motor-cognitive dual-task paradigms (Brauer et al., 2001; Plummer-D’Amato et al., 2011). Older people with poorer postural control need to prioritize their balance to avoid falling and thus cannot shift their attention to a secondary task as well as younger people or older adults with good balance (Shumway-Cook and Woollacott, 2000), a phenomenon that has been termed the “posture-first strategy.” While the number of trials required to shift the first set was a predictor for falls in the poor balance group, this was not so in the good balance group. In fact, the effect tended in the opposite direction, further supporting the idea of a protective effect of higher levels of mental flexibility on the risk of falling. Hence, it may be that preserved mental flexibility can compensate partially for reduced balance ability, but if both are impaired fall risk increases substantially. This finding is consistent with the study by Holtzer et al. (2012), who found executive function provided a protective effect against a decline in gait speed in older people with a higher cognitive reserve. Further studies could include real life simulations to examine how poor concept formation is related to the process of falling. Such work could incorporate stressors, such as complex individual-environment interactions and anxiety as these have been shown to impair mental flexibility (Hillier et al., 2006; Han et al., 2011) as well as balance-related protective factors.

Consistent with previous reports (Lord et al., 1991; Ivers et al., 1998; de Boer et al., 2004), we found contrast sensitivity was associated with falling. Impaired contrast sensitivity is also associated with reduced stride length in older people (Duggan et al., 2017) and increased body sway, especially under conditions of reduced proprioceptive feedback, such as when standing on compliant surfaces (Lord et al., 1991). Limited evidence indicates a relationship between contrast sensitivity and cognition. Risacher et al. (2013) found contrast sensitivity became progressively worse in older people classified into healthy control, subjective cognitive complaints, mild cognitive impairment, and Alzheimer’s disease groups. Further, Anstey et al. (2006) noted contrast sensitivity was significantly associated with cognitive performance in tasks of low attentional demand (processing speed) but not in tasks requiring executive control (e.g., working memory) after controlling for processing speed, supporting the (partially) independent contributions of executive function and sensory function on fall risk in older people.

Executive functions are amenable to improvements by cognitive training in older people without cognitive impairments (Kueider et al., 2012). Furthermore, exercise can improve cognitive functioning (Gregory et al., 2013) in addition to reducing fall risk (Sherrington et al., 2017). While most research has examined aerobic training (Ludyga et al., 2016), findings from one study suggest a positive effect of resistance training on cognitive outcomes in older adults (Liu-Ambrose et al., 2012). Seated cognitive training can also improve motor functions (Verghese et al., 2010; Milman et al., 2014; Smith-Ray et al., 2015), and videogames and virtual reality interventions with high attentional switching demands may contribute to improved mental flexibility (Canty et al., 2014; Cardoso-Leite and Bavelier, 2014), as well as balance control in older adults (Schoene et al., 2014). Our findings suggest the incorporation of implicit learning tasks in interventions for older people may be beneficial to improve balance and reduce fall risk. For instance, in a cognitive-motor intervention using gamified virtual reality environments, individuals could be exposed to changing and complex, unforeseen (daily life) scenarios, during which they have to extract implicit information to anticipate and avoid postural threats in order to accrue points.

We need to acknowledge some limitations. First, participants were able to step without assistance, had no major cognitive impairments and good visual acuity. Therefore, results may not be generalizable to frailer populations. Second, all tests were done in one single session and it cannot be ruled out that fatigue played a role. However, whenever required or wished individualized breaks were taken in between tests.

## Conclusion

We found older people with impaired balance had poorer mental flexibility, particularly concept formation, compared to those with good balance. Further, impaired concept formation confounded the association between balance status and falls. Our study confirms previous findings that balance is associated with higher order cognitive functions and shows older people with both impaired balance and reduced mental flexibility are at a substantially increased risk of falling. Training interventions targeting the preservation or improvement of mental flexibility may help prevent falls in this population by improving postural stability as well as cognition.

## Ethics Statement

The study was conducted according to the Declaration of Helsinki and was approved by the University of New South Wales Human Research Ethics Committee. All participants gave written informed consent prior to data collection.

## Author Contributions

DS and SL designed the study and acquired the data. BT, DS, FP-F, SL, and WK analyzed the data. All authors interpreted the results and drafted the manuscript.

## Conflict of Interest Statement

SL acknowledges the physiological profile assessment (NeuRA FallScreen) is commercially available through Neuroscience Research Australia. The remaining authors declare that the research was conducted in the absence of any commercial or financial relationships that could be construed as a potential conflict of interest.

## Footnotes

1 http://pebl.sourceforge.net

2 www.who.int/classifications/icf/whodasii/en/
