# Supplementary material for: Mental Flexibility Influences the Association Between Poor Balance and Falls in Older People – A Secondary Analysis
Source: Front Aging Neurosci. 2019 Jun 12;11:133. doi: 10.3389/fnagi.2019.00133 (PMC6584815; doi:10.3389/fnagi.2019.00133)
Supplement: Supplementary file 1 [file Table_1.DOCX]

Table S1: Correlations (Spearman-rank) between items of computerized short-form of Wisconsin Card Sorting Test (WCST)

|  | Total correct responses | Perseverative responses | Perseverative errors | # trials needed to complete the first category | % conceptual level responses | Failure to maintain set |
| --- | --- | --- | --- | --- | --- | --- |
| Total correct responses | 1 | -.227** | -.665** | -.334** | .916** | -.268** |
| Perseverative responses |  | 1 | .690** | -.452** | -.248** | -.187** |
| Perseverative errors |  |  | 1 | -.063 | -.624** | .019 |
| # trials needed to complete the first category |  |  |  | 1 | -.263** | .317** |
| % conceptual level responses |  |  |  |  | 1 | -.204** |
| Failure to maintain set |  |  |  |  |  | 1 |

**correlations <.01 (two-tailed); N=212
